# Impact of Homocysteine as a Preconceptional Screening Factor for In Vitro Fertilization and Prevention of Miscarriage with Folic Acid Supplementation following Frozen-Thawed Embryo Transfer: A Hospital-Based Retrospective Cohort Study

**DOI:** 10.3390/nu15173730

**Published:** 2023-08-25

**Authors:** Seiji Ogawa, Kuniaki Ota, Toshifumi Takahashi, Hiroaki Yoshida

**Affiliations:** 1 Sendai ART Clinic, 206-13 Nagakecho, Miyagino, Sendai 983-0864, Japan; cfy00450@gmail.com (S.O.); hiroaki@sendai-art-cl.jp (H.Y.); 2 Fukushima Medical Center for Children and Women, Fukushima Medical University, Fukushima 960-1295, Japan; toshifumi.takahashi@gmail.com; 3 Department of Obstetrics and Gynecology, Tokyo Rosai Hospital, Tokyo 143-0013, Japan

**Keywords:** homocysteine, folic acid, preconception, fertility treatment

## Abstract

Homocysteine is an amino acid naturally produced in the body and metabolized via the methionine cycle. High homocysteine levels can increase the risk of infertility and pregnancy complications, such as preeclampsia, preterm delivery, miscarriage, and low birth weight. Preconceptional homocysteine levels may be reduced by taking folic acid supplements to reduce the risk of such complications. This cross-sectional, hospital-based study was conducted to examine the role of homocysteine in 1060 infertile women with a history of IVF/intracytoplasmic sperm injection (ICSI) failure. We analyzed whether folic acid intervention altered homocysteine levels and influenced reproductive outcome. We found that a higher homocysteine level was statistically associated with a lower fertilization rate in patients with a history of IVF/ICSI failure. There was an inverse relationship between homocysteine levels and serum 25(OH)VD, and a trend towards lower anti mullerian hormone in the group with higher homocysteine levels. This is the first interventional study to identify that folic acid supplementation improved pregnancy outcomes following freeze embryo transfer (FET) in women with a history of FET failure by monitoring the reduction in homocysteine levels. Therefore, folic acid supplementation and homocysteine level monitoring may constitute a novel intervention for improving IVF/ICSI pregnancy outcomes.

## 1. Introduction

The advent of in vitro fertilization (IVF) was a fundamental breakthrough in the treatment of infertility. However, despite improved diagnostics and developed new technology, the majority of IVF cycles remain unsuccessful [1]. As infertility is a complex multifactorial condition and the factors influencing the success rate of IVF are largely unknown, the reasons for failure are often not readily apparent. In order to improve these results, researchers have focused their investigations on several aspects relating to both the clinical characteristics of the couples being treated and the technology used in the IVF laboratories; however, the results are still controversial and/or supported by little clinical evidence [2,3]. A major potential advance in infertility treatment is recognized in the identification of modifiable factors for success.

As a modifiable factor, nutritional status during the preconception period has been shown to affect reproductive performance during both natural and assisted reproduction [4,5,6]. Several recent reports have suggested that dietary patterns prior to conception as preconception care may affect IVF outcomes such as oocyte and embryo quality, implantation and successful pregnancy completion [5,7,8]. Dietary habits, particularly folic acid, have recently received attention in this context [9]. Folic acid supplementation is important for reproductive health worldwide [10] and is known to reduce the risk of neural tube abnormalities [11] and other adverse pregnancy outcomes [12]. The World Health Organization is widely known to recommend that women consume ≥400 µg of folic acid daily during the periconceptional period (from at least 4 weeks before conception to 12 weeks after) [13,14]. Internationally, many countries declare that pre-conception folic acid intake is recommended but not successful [15]. Indeed, the prevalence of preconception users with folic acid in Japan was 8.0% [16].

Folic acid plays a key role in reproductive health because its shortage results in the elevation of homocysteine, the sulfur-containing non-essential amino acid which is formed when the essential amino acid methionine is metabolized [17], and is associated with major adverse obstetric/neonatal outcomes, including neural tube defects, placental abruption, and other congenital malformations [18,19,20]. Recently, the influence of homocysteine metabolism regulators on human reproductive physiology has been investigated. Homocysteine is present in the follicular fluid of maturing oocytes. An excess of homocysteine or a deficiency in folic acid could affect oocyte development and early embryogenesis. Therefore, improving the follicular environment through appropriate oral supplementation with folic acid is effective [21]. Furthermore, homocysteine, a marker of thrombophilia, plays a key role in the regulation of vascular function. In elevated homocysteine, so-called hyperhomocysteinemia, nitric oxide becomes saturated, and excess homocysteine induces endothelial damage, reducing nitric oxide bioavailability and S-nitrous homocysteine production [22]. As such, hyperhomocysteinemia leads to impaired vasorelaxation and antiplatelet effects [23], and is believed to be harmful for implantation to decrease sufficient blood flow at the site of implantation and placentation [24]. Elevated levels of homocysteine have been associated with an increased risk of spontaneous pregnancy loss [25,26]. Furthermore, homocysteine has been reported to be inversely associated with fertility outcomes [9], and lower levels of homocysteine have been found to correlate with an improved chance of a clinical pregnancy and with a better quality of embryos in assisted reproductive technology (ART) [27,28]. Nevertheless, little is known about the association between homocysteine levels and infertility. There are also no clear data on the role of folic acid in improving success rates in couples undergoing ART [29,30].

Therefore, in a cross-sectional, hospital-based study, we aimed to examine the association of homocysteine with 1060 infertile women with a history of IVF/intracytoplasmic sperm injection (ICSI). In addition, we analyzed whether folic acid, which has the effect of lowering homocysteine, could be used as an effective intervention in some of these patients to evaluate the variation in homocysteine levels and whether the intervention of folic acid influenced reproductive outcomes.

## 2. Materials and Methods

### 2.1. Subjects Characteristics and Data Handling

This study was a retrospective analysis of 1060 infertile women treated by IVF/ICSI who had undergone previous embryo transfer cycles that did not result in a pregnancy from 2019 to 2021 at Sendai ART clinic (Miyagi, Japan). Clinical parameters such as age, BMI (determined as weight in kg divided by height meters squared), history of pregnancy (gravity, parity, and miscarriage), lifestyle (smoking status, alcohol consumption, and exercise), and the reason for infertile factors were obtained from the medical records at the initial visit. All patients underwent a routine infertility evaluation that included basal FSH, LH, and E_2_ levels on day 3 of the menstrual cycle, and AMH, 25(OH) vitamin D, and homocysteine levels at the initial visit. Furthermore, homocysteine was analyzed as a continuous and categorical variable divided into four quartiles: ≤25th, 25th to ≤50th, 50th to ≤75th, and >75th percentiles.

Patients were excluded if they had any of the following conditions: (1) acute cardiovascular disease, renal function damage, or malignant tumor; (2) intrauterine lesions, such as submucosal fibroids and endometrial polyps, requiring surgery; or (3) severe anemia due to hypermenorrhea with uterine adenomyosis.

IVF/ICSI procedures included ovarian stimulation, oocyte retrieval, and insemination by IVF or ICSI. Depending on the clinical indication, ovarian stimulation was performed using a long regimen, short regimen, antagonist regimen, moderate stimulation, or progestin-primed ovarian stimulation (PPOS). Moderate stimulation was defined as combining clomiphene with lower doses of gonadotropins used as an effective alternative to conventional ovulation regimens in women with poor ovarian response (POR). PPOS involves the administration of a single oral dose of progestogen instead of GnRH on each day of the latency period up to the day of the trigger day to inhibit ovulation. Records of IVF/ICSI cycles were examined to collect data regarding the total gonadotropin dose, endometrial thickness on the trigger day, the level of estradiol on the trigger day, methods of fertilization (IVF, ICSI, or Split), the number of oocytes retrieved, the oocytes in the metaphase II (MII) stage, the MII rate, the fertilization rate, and the rate of high-quality blastocysts. The rate of MII was equal to the number of oocytes at MII divided by the number of oocytes retrieved. The fertilization rate was equal to the number of embryos divided by the number of oocytes retrieved for IVF or the number of MII-staged oocytes retrieved for ICSI. The rate of high-quality blastocysts was calculated as the number of high-quality embryos divided by the number of embryos.

### 2.2. Prospective Interventional Study of Folic Acid Intake

**Figure 1:**
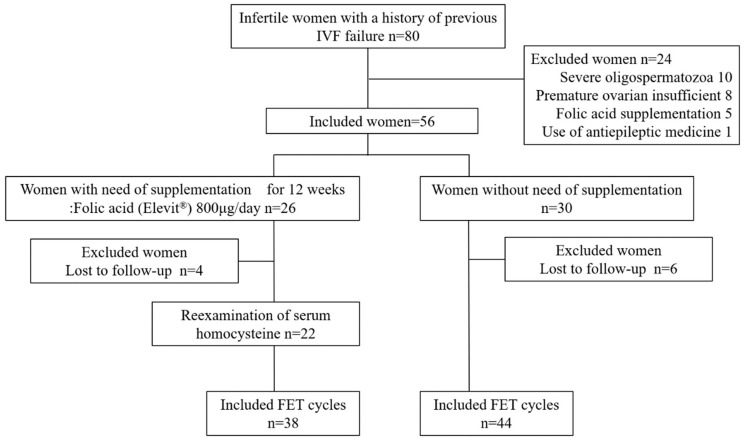
Flowchart showing patient selection. Of the 80 infertile women with a history of one or more FET failures who attended our clinic for fertility treatment between April 2019 and July 2022, 56 were recruited after excluding 24 who diagnosed severe oligospermatozoa as male factor (*n* = 10) and premature ovarian insufficient (*n* = 8) or had taken folic acid supplementation (*n* = 5) and/or medications that potentially inhibit folate absorption or conversion to its active form, including antiepileptic drugs (*n* = 1).

Of the 80 infertile women with a history of one or more FET failures who attended our clinic for fertility treatment between April 2019 and July 2022, 56 were recruited after excluding 24 who diagnosed severe oligospermatozoa as male factor (*n* = 10) and premature ovarian insufficient (*n* = 8) or had taken folic acid supplementation (*n* = 5) and/or medications that potentially inhibit folate absorption or conversion to its active form, including antiepileptic drugs (*n* = 1) [31] (Figure 1). Blood samples for analysis of serum homocysteine levels were taken before the start of this study. After the exclusion of 4 women who were lost to follow-up, 26 women received daily folic acid supplementation (Elevit^®^, Bayer Yakuhin, Ltd., Tokyo, Japan) and measured serum homocysteine levels after 12 weeks. This supplementation included 800 µg of folic acid, in addition, 1.3 mg of vitamin B1, 1.5 mg of vitamin B2, 12 mg of niacin, 1.4 mg of vitamin B6, 2.8 µg of vitamin B12, 5.0 mg of pantothenic acid, 50 µg of biotin, 100 mg of vitamin C, 7200 µg of beta-carotene, 7.0 µg of vitamin D, 6.5 mg of vitamin E, 125 mg of calcium, 100 mg of magnesium, 21.5 mg of iron, 7.5 mg of zinc, 0.9 mg of copper, and 1.0 mg of manganese are contained as well. We further examined the pregnancy outcomes as the secondary outcome, including an analysis of whether intervention with 800 μg of folic acid supplementation improved the pregnancy course until live birth following FET. Therefore, we finally compared pregnancy outcomes within 3 months between 22 women with folic acid supplementation and 24 women without supplementation. A live birth was defined as when a neonate was delivered between 37 and 41 weeks. Miscarriage was defined as the loss of clinical pregnancy, which indicated the detection of an intrauterine gestational sac by transvaginal ultrasound.

### 2.3. Ethical Statement

This study was approved by the Ethics Review Committee of Fukushima Medical University, Fukushima, Japan. Written informed consent was obtained from all participants following the provision of a detailed description of the purpose of this study. All experimental procedures were performed in accordance with the principles of the Declaration of Helsinki.

### 2.4. Statistical Analyses

Data for continuous variables were reported as the means ± SD or medians (interquartile range [IQR]) and tested by applying the single-factor analysis of variance (ANOVA) test using two-tailed tests for significance. Differences before and after folic acid supplementation were tested by the paired *t*-test, and clinical outcomes (clinical pregnancy and miscarriage rates) were assessed using Fisher‘s extract test. No statistical sample size calculations were conducted, however, a sample size of approximately 20 patients per group gave post hoc powers of 42%, 40%, and 32% to detect differences in mean of 29%, 30%, and 25%, respectively, for the clinical evaluation following FET, assuming a common SD of 33%, using a two-group *t*-test with a two-sided significance level of *p* < 0.05 for percentage change from baseline between folic acid and no supplementation. The correlation between homocysteine and 25(OH)VD was tested using Pearson’s correlation coefficient and bivariate analysis. Data were analyzed using statistical software (EZR version 1.51) [32] and all statistical tests used a significance level of *p* < 0.05.

## 3. Results

### 3.1. Baseline Characteristics of the Study Participants

The clinical characteristics of the 1060 infertile women with a history of IVF-ICSI failure included in this study are shown in Table 1. The mean age of the participants was 36.3 ± 4.4 years. Gravity, parity, and spontaneous miscarriage were 0.48 ± 0.8, 0.16 ± 0.4, and 0.16 ± 0.4, respectively. BMI measurements ranged from 14.8 to 40.9 kg/m^2^ (mean, 21.6 ± 3.5 kg/m^2^). The incidences of causal factors of infertility were uterine factor: 14.9% (*n* = 158), tubal factor: 24.0% (*n* = 255), ovulation factor: 15.8% (*n* = 164), polycystic ovarian syndrome (PSOC): 6.1% (*n* = 65), cervical factor: 9.5% (*n* = 101), endometriosis: 14.8% (*n* = 157), male factor: 32.0% (*n* = 339), and unexplained: 29.8% (*n* = 316). In terms of lifestyle, 2.3%, 3.1%, and 6.4% of participants had smoking habits including current smoker or past smoker, alcohol consumption, and exercise, respectively. Baseline levels of serum FSH, LH, and estrogen on day 3 of the menstrual cycle were 8.0 ± 3.1 IU/mL, 5.7 ± 2.6 IU/mL, and 43.5 ± 44.5 pg/mL, respectively. Mean AMH was 3.12 ± 3.0 ng/mL, and mean serum 25(OH)D level was 14.5 ± 5.8 ng/mL. Almost all the regimens for controlled ovarian stimulation were GnRH-agonists (72.2%), although histories of IVF/ICSI, IVF, ICSI, and split accounted for 43.9%, 42.7%, and 10.1%, respectively. On average, the total number of oocytes retrieved per IVF/ICSI was 7.82 ± 5.9, and the number of M-II oocytes were 6.1 ± 4.8; thus, the MII rate was 81.25. The fertilization rate was 74.0% and the number of high-quality blastocysts preserved as freeze-embryos was 1.8 ± 2.3 on average; thus, the high-quality blastocyst rate was 35.8%.

### 3.2. Association of Homocysteine Status with Reproductive Makers

**Figure 2:**
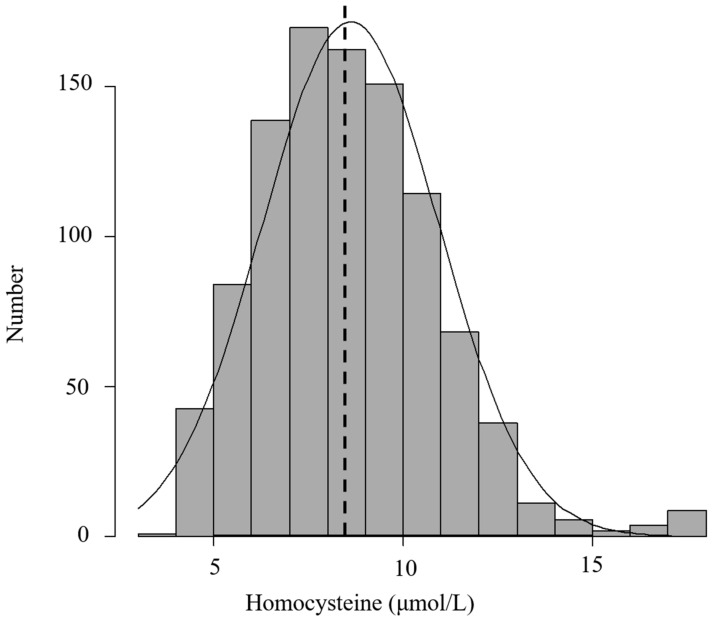
Distribution of Homocysteine in the 1060 infertile women, with a median value of 8.5 μmol/L (Dotted line).

The mean homocysteine was 8.6 ± 2.3 μmol/L, while the median was 8.5 μmol/L, with data following a normal distribution (Figure 2). Hyperhomocysteinemia has previously been defined as an elevated serum concentration of homocysteine exceeding 15 μmol/L; following this definition, the prevalence of hyperhomocysteinemia among 1060 infertile women was 1.4% (15/1060). Table 2 shows the distribution of reproductive markers and the previous history of IVF/ICSI according to the quartiles of homocysteine concentration across subjects. Each quartile of homocysteine levels was <7.0 μmol/L in the lowest quartile, ≤7.0–8.5< μmol/L in the second quartile, ≤ 8.5–10.1< μmol/L in the third quartile, and ≤ 10.1 μmol/L in the highest quartile. In terms of serum levels for Homocysteine, which were stratified into quartiles, 25(OH) vitamin D, the previous No. of IVF failure and fertilization rate reveals significant differences among 1060 infertile women who fell within the different quartiles (*p* < 0.0001, *p* < 0.0005, *p* = 0.0349, respectively). In addition, infertile women with the lowest homocysteine level (1st quartile) have a significantly higher concentration of serum 25(OH) vitamin D than those with higher homocysteine levels (2nd, 3rd, and 4th quartiles) (*p* < 0.0001), and those with the lowest homocysteine level (1st quartile) have a significantly higher fertilization rate than those with the highest homocysteine levels (4th quartiles). No significant between these quartiles were found in AMH, basal FSH, basal LH, basal E2, or previous number of ET failures, E2 on hCG trigger, total FSH/HMG amount, number of retrieved oocytes, number of MII, MII rate, number of blastocysts, and the high-quality blastocyst rate.

### 3.3. Impact of Folic Acid Supplementation on Homocysteine Levels and Pregnancy Outcome

**Figure 3:**
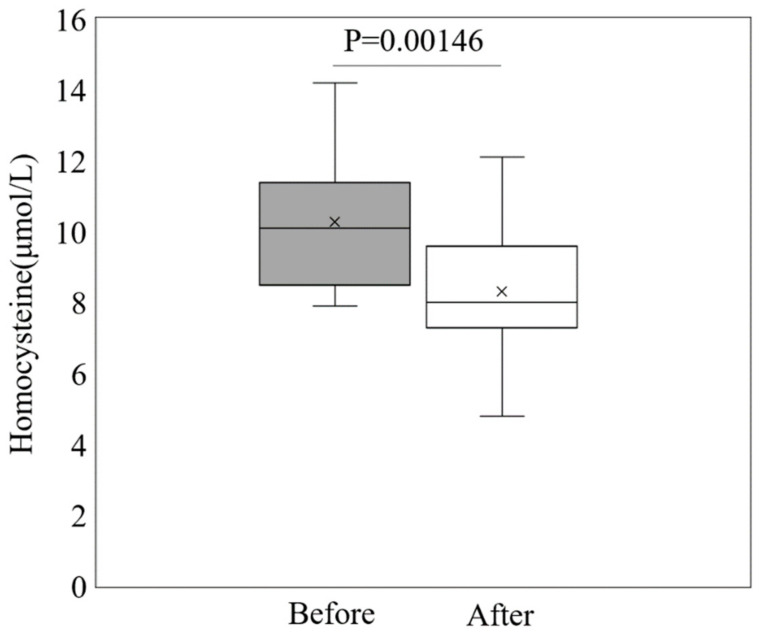
Changes in the homocysteine level before and after folic acid supplementation in infertile women with a history of FET failures.

The clinical characteristics of the 46 infertile women with a history of one or more FET failures included in this prospective interventional study are shown in Table 3. The 25(OH) vitamin D levels in women receiving folic acid supplementation were lower than those in women without folic acid supplementation, while homocysteine levels were higher. The other parameters were not significantly different between the folic acid supplementation and no supplementation groups. We assessed serum homocysteine levels in 23 women before and after folic acid supplementation to determine the effects of folic acid intervention on homocysteine levels in infertile women with a history of one or more FET failures. Folic acid supplementation for 12 weeks significantly decreased serum homocysteine levels (*p* = 0.00146, Figure 3). In addition, to identify the effects of folic acid supplementation on pregnancy outcome (clinical pregnancy and miscarriage rate) following FET, miscarriage was statistically prevented by folic acid supplementation (95% CI: 0.02–1.20; *p* < 0.05), although clinical pregnancy rates did not improve with folic acid supplementation (95% CI: 0.15–1.25; *p* = 0.97) (Table 4).

## 4. Discussion

In the present study, we showed that higher homocysteine levels are associated with significantly lower fertilization rates and vitamin D levels. We further observed a trend towards lower AMH levels in the group with higher homocysteine levels. This is the first study to show that folic acid supplementation significantly lowers homocysteine levels and significantly improves the rate of miscarriage following FET in women with a history of FET failure.

It is true that the exact pathogenesis of unexplained infertility is not yet fully understood [33,34]; Liu et al. recently reported that there was a negative correlation between serum homocysteine and the total number of oocytes retrieved using the Spearman correlation analysis and univariate linear regression analysis [35], although there was no statistical correlation between serum homocysteine and the number of oocytes retrieved in this study. In addition, those mechanisms were unknown although previous studies have shown that a high concentration of serum homocysteine may lead to a significant reduction in the number of retrieved oocytes and M-II oocytes, suppress fertilization rate, and reduce the number of high-quality embryos following IVF/ICSI treatment [27,28,36,37]. Homocysteine is known to have a common biochemical basis as the non-specific induction of oxidative stress, and a high level of homocysteine can be responsible for the toxic effect. Oyawoye et al. [38] found that the anti-potency against oxidative stress in follicular fluid was associated with successfully fertilized oocytes compared to non-fertilized oocytes. Interestingly, Ocal et al. [28] revealed that a high homocysteine level in follicular fluid is associated with reducing the rate of clinical pregnancy. Hence, focal homocysteine in follicular fluid may be harmful to oocytes in the ovaries, although it raised the possibility that focal homocysteine in follicular fluid may have reflected systemic homocysteine due to increased vascular permeability in granulosa cells in the ovary after HCG triggering. Our findings are consistent with previous reports that higher levels of serum homocysteine worsen the fertilization rate, although other parameters such as the number of oocysts retrieved and MII oocytes were not associated with the level of homocysteine. Furthermore, a recent retrospective cohort study reported that serum homocysteine levels were statistically lower in patients with high-quality embryos than in those with poor-quality embryos, and only serum homocysteine levels were found to correlate with embryo quality in a logistic regression analysis [39]. Our results were consistent with a statistically significant decrease in AMH levels in the group with higher homocysteine levels; however, there was no association between AMH, homocysteine, and embryo quality. Although the study size and design were the same, different results were obtained as this prior study focused only on diminished ovarian reserve, which might affect embryo quality.

The status in women with the low levels of serum 25(OH)D is a major global health problem, and most women in the preconception period have been at risk of vitamin D deficiency/insufficiency [40,41,42]. In particular, vitamin D deficiency is traditionally associated with maternal and neonatal bone disease, however, it is more prevalent in women with major reproductive disorders [43,44]. The impact of vitamin D in maintaining pregnancy has been reported in studies of IVF [45]. It is also estimated that 31% of pregnancies end in miscarriage, with two-thirds of miscarriages being clinically undetectable as to vitamin D deficiency/insufficiency [46]. Studies have shown that women who experienced normal pregnancies and delivered without perinatal troubles had significantly higher vitamin D levels than those who had a history of miscarriages [47]. We previously reported that women with recurrent miscarriages and low vitamin D levels were more likely to develop autoimmune and cellular immunological disorders than women with recurrent miscarriages and normal vitamin D levels [48,49,50]. In this study, only nine women were found to be vitamin D sufficiency (>30 ng/mL), with 0.8% (*n* = 1051) being vitamin D deficiency/insufficiency. Hence, vitamin D deficiency/insufficiency may have caused reproductive abnormalities in this study. In this interventional study, 7.0 μg of vitamin D per day was also included as well as folic acid, so it is possible that a preventive effect of vitamin D on miscarriage may have been speculated. On the other hand, pregnant women who consumed less than 10 µg of vitamin D per day were found to be most likely to miscarriage [51]; therefore, we speculated that 800 µg of folic acid per day may play a pivotal role in to preventing miscarriage compared to 7.0 µg of vitamin D per day in this study.

**Figure 4:**
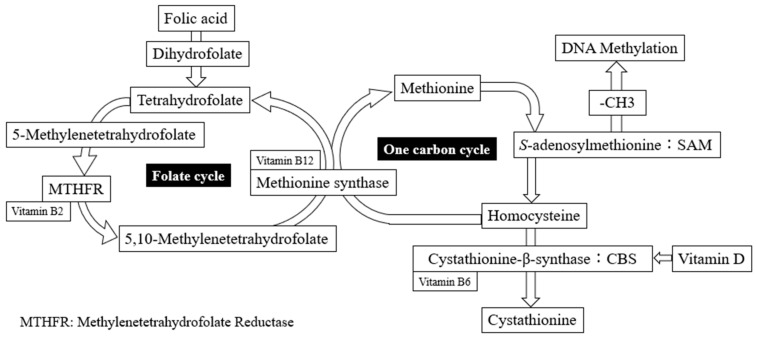
The one-carbon and the folates cycles. Synthetic folic acid must undergo two metabolic steps to enter the folate cycle.

Vitamin D has been shown to act as a co-factor in homocysteine metabolism by directly regulating the levels of cystathionine β-synthase which is an enzyme involved in homocysteine degradation, and resulted in the conversion from homocysteine to cystathionine and cysteine as transsulfuration pathway products [52]. Interestingly, both large nationwide cohort studies from the Longitudinal Aging Study Amsterdam in the Netherlands and the National Health and Nutrition Examination Survey (NHANES) in the United States showed an inverse relationship between 25(OH)D and homocysteine among healthy adults with 25(OH)D concentrations since vitamin D may counteract the impact of high homocysteine levels by increasing cystathionine-β-synthase synthesis and enzymatic activity [52,53] (Figure 4). Previously, we similarly described an inverse relationship between homocysteine levels and serum 25(OH)VD in women with RPL [54]. We speculate that the etiology of reproductive failure, such as recurrent implantation failure and RPL, based on the fact that the coexistence of low vitamin D and high homocysteine levels may lead to an altered inflammatory response, predisposing to microvascular and endothelial damage. In this study, higher homocysteine levels were found to be associated with lower 25(OH)VD levels, although there was no negative correlation between homocysteine and 25(OH)VD (r = −0.178, *p* < 0.00001) (Supplement Figure S1). Furthermore, it has raised the possibility that vitamin D treatment may reduce homocysteine concentration and result in preventing reproductive failures such as miscarriage. Concretely, Al-bayyari et al. [55] reported that vitamin D3 intervention at the treatment dose of 50,000 IU per week for at least 2 months can help in decreasing homocysteine levels among reproductive women. In this interventional study, this supplementation contained only 7 μg of vitamin D per day, so it seems unlikely that it specifically lowered homocysteine to prevent miscarriage. Hence, we currently considered that 800 μg folic acid per day was superior to reducing homocysteine concentration and resulting in preventing miscarriage.

The effect of folic acid supplementation on lowering serum homocysteine levels in various disease states is well established, as folic acid is indirectly converted from homocysteine to methionine in one-carbon metabolism, resulting in the reduction in serum homocysteine [56] (Figure 4). Elevated maternal homocysteine can directly damage endothelial cells and affect placental perfusion and function, leading to higher pregnancy loss rates [57]. Therefore, some researchers have reported that preconceptional elevated homocysteine levels are linked with pregnancy loss [58,59,60] since Steegers-Theunissen et al. [18] were the first to publish on the relationship between high homocysteine levels and spontaneous pregnancy loss before the first 20 weeks of gestation. Moreover, it is tempting to speculate that lowering homocysteine and folic acid levels in women could decrease pregnancy loss, as folic acid supplementation of 0.5–5.0 mg can lower homocysteine levels by 25% [61]. A recent prospective study described that >730 μg/day of folic acid supplementation was associated with reduced spontaneous abortion in multivariable analysis (relative risk:0.80 95% CI 0.71–0.90) [62]. Previously, a randomised controlled trial for ART patients with hyperhomocysteine reported that the intervention with 400 μg of folic acid improved pregnancy rate and miscarriage rate [63]. However, this population with hyperhomocysteimia was defined more than 17.0 μmol/L, and there were nine cases with more than 17.0 μmol/L (0.08%) in this study [63]. In the present study, 800 μg/day of folic acid supplementation for 12 weeks significantly decreased homocysteine levels and consequently prevented miscarriage following FET. We speculated that folic acid supplementation might decrease homocysteine, which can cause endothelial cell damage and improve placental blood vessels, to successfully promote ongoing pregnancy. To our knowledge, this is the first interventional study of folic acid in the IVF/ICSI program to reduce the miscarriage rate while monitoring serum homocysteine levels. In addition, a recent report described that miscarriages were recurrent in the next pregnancy if women with a history of miscarriages were HCY > 10 μmol/L [64]. In this interventional study, the mean of homocysteine in which 800 μg of folic acid prevented miscarriage was 10.1 μmol/L, which is consistent with their study. Numerous associations between homocysteine and miscarriage have been reported [65], although the actual values for the treated population are unknown. Taken together, if homocysteine levels were above 10, therapeutic intervention seems warranted to prevent miscarriage. However, it is not clear whether 800 μg of folic acid would be as effective for miscarriages as the intervention we used. More probable may be that the administration of low-molecular-weight heparin will improve uterine artery flow and pregnancy outcome in women with recurrent miscarriage, because homocysteine may be involved in thrombosis by inducing oxidative stress, which may reduce blood flow to the implantation site and placenta.

Despite these strengths, the current study had some limitations. First, this was a single-center, retrospective study. Second, the sample size in the subgroup analysis for folic acid supplementation was relatively small, and there was no control group, such as a placebo. Third, dietary assessment was not conducted; therefore, the effects of dietary folate could not be analyzed. Fourth, homocysteine is also regulated by genetic factors, such as MTHFR (methylenetetrahydrofolate reductase) and vitamin B, which were not assessed in this study. Further large, prospective, and multicenter studies are warranted to validate the findings of this study. Nevertheless, some strengths of this study are worth noting. Most notably, this study was based on the largest sample representative of infertile Japanese women who underwent measurement of serum homocysteine as preconceptional screening.

## 5. Conclusions

In conclusion, we found that higher homocysteine levels were significantly associated with lower fertilization rates in patients with a history of IVF/ICSI failure. Furthermore, we found an inverse relationship between homocysteine levels and serum 25(OH)VD and observed a trend toward lower AMH in the group with higher homocysteine levels. This is the first interventional study to identify that folic acid supplementation improved pregnancy outcomes following FET in women with a history of FET failure by monitoring the reduction in homocysteine levels. Taken together, we suggest that folic acid supplementation to monitor homocysteine levels may be a novel intervention for the improvement of IVF/ICSI pregnancy outcomes. Further large-scale prospective studies to examine the association between homocysteine levels and folic acid supplementation and IVF/ICSI pregnancy outcomes are warranted to support our findings.

## Figures and Tables

**Table 1:** Patient characteristics.

Number	1060
Age(y)	36.3 ± 4.4
Gravity	0.48 ± 0.8
Parity	0.16 ± 0.4
Miscarriage	0.32 ± 0.6
BMI (kg/m^2^)	21.6 ± 5.4
Type of infertility	
Uterine factor	14.9% (158)
Fallopian tube factor	24.0% (255)
Ovulation factor	15.8% (164)
PCOS	6.1% (65)
Cervical factor	9.5% (101)
Endometriosis	14.8% (157)
Male factor	32.0% (339)
unexplained	29.8% (316)
Life style	
Smoking	2.3% (24)
Alcohol	3.1% (33)
Exercise	6.4% (68)
Basal FSH (IU/mL)	8.0 ± 3.1
Basal LH(IU/mL)	5.7 ± 2.6
Basal E2 (pg/mL)	43.5 ± 44.5
AMH (ng/L)	3.12 ± 3.0
25(OH) vitamin D (ng/mL)	14.5 ± 5.8
Serum Homocysteine level (μmol/L)	8.6 ± 2.3
Previous No. of IVF/ICSI failure	1.9 ± 1.5
Previous No. of ET failure	0.6 ± 1.1
Total gonadotropin dose used (IU/L)	1647.2 ± 619.8
Endometrial thickness on hCG day (mm)	10.1 ± 2.8
E2 level on hCG day(pmol/L)	2042.6 ± 1386.2
Fertilization	
IVF	43.9% (465)
ICSI	42.7% (453)
Split	10.1% (107)
Ovulation regimens	
GnRH-agonist long protocol	1.0% (11)
GnRH-agonist short protocol	0.6% (7)
GnRH-antagonist	72.2% (765)
Moderate stimulation	8.5% (90)
PPOS	17.6% (187)
Total number of oocytes retrieved	7.82 ± 5.9
No. of M-II oocytes	6.1 ± 4.8
MII rate	81.2%
Fertilization rate	74.0%
No. of high-quality blastocyst	1.8 ± 2.3
High quality blastocyst rate	35.8%

**Table 2:** Clinical characteristics of infertile women with a history of FET failure according to homocysteine levels.

	Quartile of Homocysteine Level, μmol/L				
	Q1 (<7.0)	Q2 (≤7.0–8.5<)	Q3 (≤8.5–10.1<)	Q4 (≥10.1)	*p*
AMH	3.27 ± 3.1	3.52 ± 3.3	2.82 ± 2.5 *	3.13 ± 2.9	0.05
Basal FSH	7.76 ± 3.0	8.05 ± 3.1	7.90 ± 3.3	8.41 ± 3.2	0.11
Basal LH	5.62 ± 2.8	5.52 ± 2.5	5.52 ± 2.5	5.93 ± 2.8	0.34
Basal E2	39.8 ± 28.5	37.9 ± 21.1	47.1 ± 77.2	44.0 ± 23.0	0.09
25(OH)VD	16.3 ± 5.7 **	14.1 ± 6.0	14.0 ± 6.1	13.2 ± 5.2	<0.001
Previous No. of IVF failure	1.7 ± 1.2	1.9 ± 1.5	1.9 ± 1.2	2.3 ± 2.0	<0.001
Previous No. of ET failure	0.6 ± 1.1	0.5 ± 1.1	0.7 ± 1.2	0.48 ± 1.1	0.21
E2 on hCG trigger	2232.3 ± 1505.8	2008.4 ± 1396.1	1925.0 ± 1386.0	2004.5 ± 1233.0	0.07
Total FSH/HMG amount	1685.2 ± 588.4	1604.7 ± 587.7	1675.5 ± 712.4	1623.9 ± 580.7	0.37
No. of oocytes retrieved	8.1 ± 6.3	7.6 ± 5.6	7.6 ± 5.9	8.0 ± 6.0	0.68
No. of MII	6.4 ± 5.3	5.9 ± 4.4	6.0 ± 5.0	6.2 ± 4.6	0.68
MII rate (%)	80.9 ± 21.3	81.8 ± 20.4	82.2 ± 22.0	79.9 ± 23.5	0.62
Fertilization rate (%)	75.6 ± 26.4	72.7 ± 27.4	76.8 ± 24.0	70.7 ± 26.1 ***	0.03
No. of Blastocyst	1.8 ± 2.5	1.8 ± 2.3	1.7 ± 2.1	1.9 ± 2.2	0.86
High-quality blastocyst rate (%)	34.9 ± 32.2	35.5 ± 32.9	36.5 ± 33.4	36.2 ± 31.0	0.95

*: Q2 VS Q3, *p* < 0.05; **: Q1 VS Q2,Q3,Q4, *p* < 0.0001; ***: Q1 VS Q4, *p* < 0.01.

**Table 3:** Comparison of clinical features between the folic acid supplementation and no supplementation groups.

	Folic Acid (*n* = 22)	No Intervention (*n* = 24)	*p* Value
Age(y)	34.2 ± 3.0	35.0 ± 4.3	0.56
BMI	21.8 (18.9–23.2)	21.6 (19.6–24.0)	0.67
Gravity	0 (0–0)	0 (0–1)	0.18
Parity	0 (0–0)	0 (0–0)	0.64
Miscarriage	0 (0–0)	0 (0–1)	0.24
Basal FSH (IU/mL)	6.4 ± 1.9	7.2 ± 2.3	0.26
Basal LH (IU/mL)	5.0 ± 2.1	5.7 ± 2.6	0.34
Basal E2 (pg/ml)	38.0 (25.0–51.0)	39.0 (25.0–50.3)	0.85
25(OH)vitamin (ng/mL)	10.8 ± 5.0	14.5 ± 4.6	0.01
AMH (ng/mL)	2.45 (1.34–4.95)	2.80 (1.47–4.67)	0.10
Homocysteine (μmol/L)	10.1 (8.6–11.4)	8.6 (7.8–9.6)	0.01

**Table 4:** Clinical evaluation of pregnancy outcome following FET after folic acid supplementation.

	Folic Acid (*n* = 22, 38 FET cycles)	No Supplementation (*n* = 24, 44 FET Cycles)	95% Confident Interval		*p* Value
			Low	High	
Clinical pregnancy	16 (42.1)	10 (22.7)	0.15	1.25	0.97
Miscarriage	5 (31.3)	7 (70.0)	0.02	1.20	<0.05

## Data Availability

The datasets generated and/or analyzed during this current study are not publicly available due to participant privacy, but they are available from the corresponding author on reasonable request.

## Supplementary Materials

The following supporting information can be downloaded at https://www.mdpi.com/article/10.3390/nu15173730/s1, Figure S1: The association with homocysteine and 25(OH)VD.
