# Supplementary figures and images for: Impact of Homocysteine as a Preconceptional Screening Factor for In Vitro Fertilization and Prevention of Miscarriage with Folic Acid Supplementation following Frozen-Thawed Embryo Transfer: A Hospital-Based Retrospective Cohort Study
Source: Nutrients. 2023 Aug 25;15(17):3730. doi: 10.3390/nu15173730 (PMC10490052; doi:10.3390/nu15173730)

## Slide 1
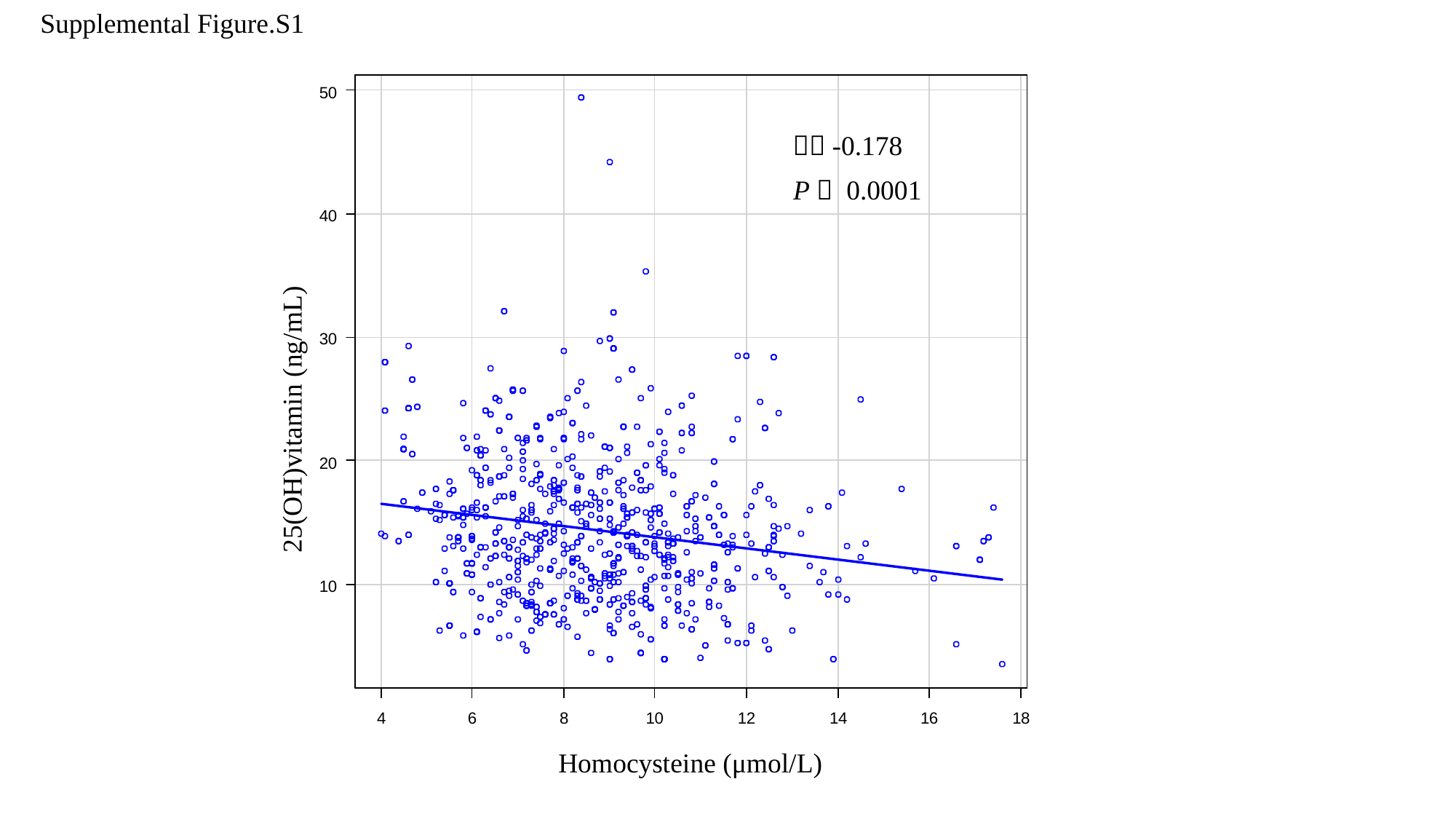

Supplemental Figure.S1
ｒ＝-0.178
P＜ 0.0001
25(OH)vitamin (ng/mL)
Homocysteine (μmol/L)

Supplement: Supplementary file 1 [file nutrients-15-03730-s001.zip › nutrients-2478144-supplementary.pptx]
